# A Quick Guide to Organizing Computational Biology Projects

**DOI:** 10.1371/journal.pcbi.1000424

**Published:** 2009-07-31

**Authors:** William Stafford Noble

**Affiliations:** 1 Department of Genome Sciences, School of Medicine, University of Washington, Seattle, Washington, United States of America; 2 Department of Computer Science and Engineering, University of Washington, Seattle, Washington, United States of America; Whitehead Institute, United States of America

## Introduction

Most bioinformatics coursework focuses on algorithms, with perhaps some components devoted to learning programming skills and learning how to use existing bioinformatics software. Unfortunately, for students who are preparing for a research career, this type of curriculum fails to address many of the day-to-day organizational challenges associated with performing computational experiments. In practice, the principles behind organizing and documenting computational experiments are often learned on the fly, and this learning is strongly influenced by personal predilections as well as by chance interactions with collaborators or colleagues.

The purpose of this article is to describe one good strategy for carrying out computational experiments. I will not describe profound issues such as how to formulate hypotheses, design experiments, or draw conclusions. Rather, I will focus on relatively mundane issues such as organizing files and directories and documenting progress. These issues are important because poor organizational choices can lead to significantly slower research progress. I do not claim that the strategies I outline here are optimal. These are simply the principles and practices that I have developed over 12 years of bioinformatics research, augmented with various suggestions from other researchers with whom I have discussed these issues.

## Principles

The core guiding principle is simple: Someone unfamiliar with your project should be able to look at your computer files and understand in detail what you did and why. This “someone” could be any of a variety of people: someone who read your published article and wants to try to reproduce your work, a collaborator who wants to understand the details of your experiments, a future student working in your lab who wants to extend your work after you have moved on to a new job, your research advisor, who may be interested in understanding your work or who may be evaluating your research skills. Most commonly, however, that “someone” is you. A few months from now, you may not remember what you were up to when you created a particular set of files, or you may not remember what conclusions you drew. You will either have to then spend time reconstructing your previous experiments or lose whatever insights you gained from those experiments.

This leads to the second principle, which is actually more like a version of Murphy's Law: Everything you do, you will probably have to do over again. Inevitably, you will discover some flaw in your initial preparation of the data being analyzed, or you will get access to new data, or you will decide that your parameterization of a particular model was not broad enough. This means that the experiment you did last week, or even the set of experiments you've been working on over the past month, will probably need to be redone. If you have organized and documented your work clearly, then repeating the experiment with the new data or the new parameterization will be much, much easier.

To see how these two principles are applied in practice, let's begin by considering the organization of directories and files with respect to a particular project.

## File and Directory Organization

When you begin a new project, you will need to decide upon some organizational structure for the relevant directories. It is generally a good idea to store all of the files relevant to one project under a common root directory. The exception to this rule is source code or scripts that are used in multiple projects. Each such program might have a project directory of its own.

**Figure 1:**
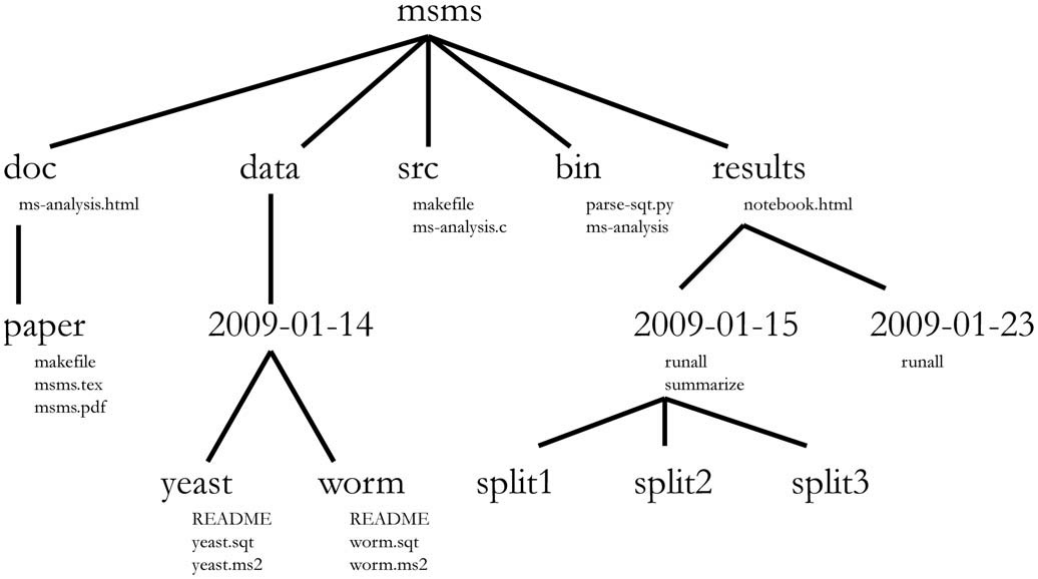
Directory structure for a sample project. Directory names are in large typeface, and filenames are in smaller typeface. Only a subset of the files are shown here. Note that the dates are formatted <year>-<month>-<day> so that they can be sorted in chronological order. The source code src/ms-analysis.c is compiled to create bin/ms-analysis and is documented in doc/ms-analysis.html. The README files in the data directories specify who downloaded the data files from what URL on what date. The driver script results/2009-01-15/runall automatically generates the three subdirectories split1, split2, and split3, corresponding to three cross-validation splits. The bin/parse-sqt.py script is called by both of the runall driver scripts.

Within a given project, I use a top-level organization that is logical, with chronological organization at the next level, and logical organization below that. A sample project, called msms, is shown in Figure 1. At the root of most of my projects, I have a data directory for storing fixed data sets, a results directory for tracking computational experiments peformed on that data, a doc directory with one subdirectory per manuscript, and directories such as src for source code and bin for compiled binaries or scripts.

Within the data and results directories, it is often tempting to apply a similar, logical organization. For example, you may have two or three data sets against which you plan to benchmark your algorithms, so you could create one directory for each of them under data. In my experience, this approach is risky, because the logical structure of your final set of experiments may look drastically different from the form you initially designed. This is particularly true under the results directory, where you may not even know in advance what kinds of experiments you will need to perform. If you try to give your directories logical names, you may end up with a very long list of directories with names that, six months from now, you no longer know how to interpret.

Instead, I have found that organizing my data and results directories chronologically makes the most sense. Indeed, with this approach, the distinction between data and results may not be useful. Instead, one could imagine a top-level directory called something like experiments, with subdirectories with names like 2008-12-19. Optionally, the directory name might also include a word or two indicating the topic of the experiment therein. In practice, a single experiment will often require more than one day of work, and so you may end up working a few days or more before creating a new subdirectory. Later, when you or someone else wants to know what you did, the chronological structure of your work will be self-evident.

Below a single experiment directory, the organization of files and directories is logical, and depends upon the structure of your experiment. In many simple experiments, you can keep all of your files in the current directory. If you start creating lots of files, then you should introduce some directory structure to store files of different types. This directory structure will typically be generated automatically from a driver script, as discussed below.

## The Lab Notebook

In parallel with this chronological directory structure, I find it useful to maintain a chronologically organized lab notebook. This is a document that resides in the root of the results directory and that records your progress in detail. Entries in the notebook should be dated, and they should be relatively verbose, with links or embedded images or tables displaying the results of the experiments that you performed. In addition to describing precisely what you did, the notebook should record your observations, conclusions, and ideas for future work. Particularly when an experiment turns out badly, it is tempting simply to link the final plot or table of results and start a new experiment. Before doing that, it is important to document how you know the experiment failed, since the interpretation of your results may not be obvious to someone else reading your lab notebook.

In addition to the primary text describing your experiments, it is often valuable to transcribe notes from conversations as well as e-mail text into the lab notebook. These types of entries provide a complete picture of the development of the project over time.

In practice, I ask members of my research group to put their lab notebooks online, behind password protection if necessary. When I meet with a member of my lab or a project team, we can refer to the online lab notebook, focusing on the current entry but scrolling up to previous entries as necessary. The URL can also be provided to remote collaborators to give them status updates on the project.

Note that if you would rather not create your own “home-brew” electronic notebook, several alternatives are available. For example, a variety of commercial software systems have been created to help scientists create and maintain electronic lab notebooks [1]–[3]. Furthermore, especially in the context of collaborations, storing the lab notebook on a wiki-based system or on a blog site may be appealing.

## Carrying Out a Single Experiment

You have now created your directory structure, and you have created a directory for the current data, with the intention of carrying out a particular experiment in that directory. How do you proceed?

The general principle is that you should record every operation that you perform, and make those operations as transparent and reproducible as possible. In practice, this means that I create either a README file, in which I store every command line that I used while performing the experiment, or a driver script (I usually call this runall) that carries out the entire experiment automatically. The choices that you make at this point will depend strongly upon what development environment you prefer. If you are working in a language such as Matlab or R, you may be able to store everything as a script in that language. If you are using compiled code, then you will need to store the command lines separately. Personally, I work in a combination of shell scripts, Python, and C. The appropriate mix of these three languages depends upon the complexity of the experiment. Whatever you decide, you should end up with a file that is parallel to the lab notebook entry. The lab notebook contains a prose description of the experiment, whereas the driver script contains all the gory details.

Here are some rules of thumb that I try to follow when developing the driver script:

1. Record every operation that you perform.
2. Comment generously. The driver script typically involves little in the way of complicated logic, but often invokes various scripts that you have written, as well as a possibly eclectic collection of Unix utilities. Hence, for this type of script, a reasonable rule of thumb is that someone should be able to understand what you are doing solely from reading the comments. Note that I am refraining from advocating a particular mode of commenting for compiled code or more complex scripts—there are many schools of thought on the correct way to write such comments.
3. Avoid editing intermediate files by hand. Doing so means that your script will only be semi-automatic, because the next time you run the experiment, you will have to redo the editing operation. Many simple editing operations can be performed using standard Unix utilities such as sed, awk, grep, head, tail, sort, cut, and paste.
4. Store all file and directory names in this script. If the driver script calls other scripts or functions, then files and directory names should be passed from the driver script to these auxiliary scripts. Forcing all of the file and directory names to reside in one place makes it much easier to keep track of and modify the organization of your output files.
5. Use relative pathnames to access other files within the same project. If you use absolute pathnames, then your script will not work for people who check out a copy of your project in their local directories (see “The Value of Version Control” below).
6. Make the script restartable. I find it useful to embed long-running steps of the experiment in a loop of the form if (<output file does not exist>) then <perform operation>. If I want to rerun selected parts of the experiment, then I can delete the corresponding output files.

For experiments that take a long time to run, I find it useful to be able to obtain a summary of the experiment's progress thus far. In these cases, I create two driver scripts, one to run the experiment (runall) and one to summarize the results (summarize). The final line of runall calls summarize, which in turn creates a plot, table, or HTML page that summarizes the results of the experiment. The summarize script is written in such a way that it can interpret a partially completed experiment, showing how much of the computation has been performed thus far.

## Handling and Preventing Errors

During the development of a complicated set of experiments, you will introduce errors into your code. Such errors are inevitable, but they are particularly problematic if they are difficult to track down or, worse, if you don't know about them and hence draw invalid conclusions from your experiment. Here are three suggestions for error handling.

First, write robust code to detect errors. Even in a simple script, you should check for bogus parameters, invalid input, etc. Whenever possible, use robust library functions to read standard file formats rather than writing ad hoc parsers.

Second, when an error does occur, abort. I typically have my program print a message to standard error and then exit with a non-zero exit status. Such behavior might seem like it makes your program brittle; however, if you try to skip over the problematic case and continue on to the next step in the experiment, you run the risk that you will never notice the error. A corollary of this rule is that your code should always check the return codes of commands executed and functions called, and abort when a failure is observed.

Third, whenever possible, create each output file using a temporary name, and then rename the file after it is complete. This allows you to easily make your scripts restartable and, more importantly, prevents partial results from being mistaken for full results.

## Command Lines versus Scripts versus Programs

The design question that you will face most often as you formulate and execute a series of computational experiments is how much effort to put into software engineering. Depending upon your temperament, you may be tempted to execute a quick series of commands in order to test your hypothesis immediately, or you may be tempted to over-engineer your programs to carry out your experiment in a pleasingly automatic fashion. In practice, I find that a happy medium between these two often involves iterative improvement of scripts. An initial script is designed with minimal functionality and without the ability to restart in the middle of partially completed experiments. As the functionality of the script expands and the script is used more often, it may need to be broken into several scripts, or it may get “upgraded” from a simple shell script to Python, or, if memory or computational demands are too high, from Python to C or a mix thereof.

In practice, therefore, the scripts that I write tend to fall into these four categories:

1. **Driver script.** This is a top-level script; hence, each directory contains only one or two scripts of this type.
2. **Single-use script.** This is a simple script designed for a single use. For example, the script might convert an arbitrarily formatted file associated with this project into a format used by some of your existing scripts. This type of script resides in the same directory as the driver script that calls it.
3. **Project-specific script.** This type of script provides a generic functionality used by multiple experiments within the given project. I typically store such scripts in a directory immediately below the project root directory (e.g., the msms/bin/parse-sqt.py file in Figure 1).
4. **Multi-project script.** Some functionality is generic enough to be useful across many projects. I maintain a set of these generic scripts, which perform functions such as extracting specified sequences from a FASTA file, generating an ROC curve, splitting a file for *n*-fold cross-validation, etc.

Regardless of how general a script is supposed to be, it should have a clearly documented interface. In particular, every script or program, no matter how simple, should be able to produce a fairly detailed usage statement that makes it clear what the inputs and outputs are and what options are available.

## The Value of Version Control

Version control software was originally developed to maintain and coordinate the development of complex software engineering projects. Modern version control systems such as Subversion are based on a central repository that stores all versions of a given collection of related files. Multiple individuals can “check out” a working copy of these files into their local directories, make changes, and then check the changes back into the central repository.

I find version control software to be invaluable for managing computational experiments, for three reasons. First, the software provides a form of backup. Although our university computer systems are automatically backed up on a nightly basis, my laptop's backup schedule is more erratic. Furthermore, after mistakenly overwriting a file, it is often easier to retrieve yesterday's version from Subversion than to send an e-mail to the system administator. Indeed, one of my graduate students told me he would breathe a sigh of relief after typing svn commit, because that command stores a snapshot of his working directory in the central repository.

Second, version control provides a historical record that can be useful for tracking down bugs or understanding old results. Typically, a script or program will evolve throughout the course of a project. Rather than storing many copies of the script with slightly different names, I rely upon the version control system to keep track of those versions. If I need to reproduce exactly an experiment that I performed three months ago, I can use the version control software to check out a copy of the state of my project at that time. Note that most version control software can also assign a logical “tag” to a particular state of the repository, allowing you to easily retrieve that state later.

Third, and perhaps most significantly, version control is invaluable for collaborative projects. The repository allows collaborators to work simultaneously on a collection of files, including scripts, documentation, or a draft manuscript. If two individuals edit the same file in parallel, then the version control software will automatically merge the two versions and flag lines that were edited by both people. It is not uncommon, in the hours before a looming deadline, for me to talk by phone with a remote collaborator while we both edit the same document, checking in changes every few minutes.

Although the basic idea of version control software seems straightforward, using a system such as Subversion effectively requires some discipline. First, version control software is most useful when it is used regularly. A good rule of thumb is that changes should be checked in at least once a day. This ensures that your historical record is complete and that a recent backup is always available if you mistakenly overwrite a file. If you are in the midst of editing code, and you have caused a once-compilable program to no longer work, it is possible to check in your changes on a “branch” of the project, effectively stating that this is a work in progress. Once the new functionality is implemented, then the branch can be merged back into the “trunk” of the project. Only then will your changes be propagated to other members of the project team.

Second, version control should only be used for files that you edit by hand. Automatically generated files, whether they are compiled programs or the results of a computational experiment, do not belong under version control. These files tend to be large, so checking them into the project wastes disk space, both because they will be duplicated in the repository and in every working copy of the project, and also because these files will tend to change as you redo your experiment multiple times. Binary files are particularly wasteful: Because version control software operates on a line-by-line basis, the version history of a binary file is simply a complete copy of all versions of that file. There are exceptions to this rule, such as relatively small data files that will not change through the experiment, but these exceptions are rare.

One practical difficulty with not checking in automatically generated files is that each time you issue an update command, the version control software is likely to complain about all of these files in your working directory that have not been checked in. To avoid scrolling through multiple screens of filenames at each update, Subversion and CVS provide functionality to tell the system to ignore certain files or types of files.

## Conclusion

Many of the ideas outlined above have been described previously either in the context of computational biology or in general scientific computation. In particular, much has been written about the need to adopt sound software engineering principles and practices in the context of scientific software development. For example, Baxter et al. [4] propose a set of five “best practices” for scientific software projects, and Wilson [5] describes a variety of standard software engineering tools that can be used to make a computational scientist's life easier.

Although many practical issues described above apply generally to any type of scientific computational research, working with biologists and biological data does present some of its own issues. For example, many biological data sets are stored in central data repositories. Basic record keeping—recording in the lab notebook the URL as well as the version number and download date for a given data set—may be sufficient to track simpler data sets. But for very large or dynamic data, it may be necessary to use a more sophisticated approach. For example, Boyle et al. [6] discuss how best to manage complex data repositories in the context of a scientific research program.

In addition, the need to make results accessible to and understandable by wet lab biologists may have practical implications for how a project is managed. For example, to make the results more understandable, significant effort may need to go into the prose descriptions of experiments in the lab notebook, rather than simply including a figure or table with a few lines of text summarizing the major conclusion. More practically, differences in operating systems and software may cause logistical difficulties. For example, computer scientists may prefer to write their documents in the LaTeX typesetting language, whereas biologists may prefer Microsoft Word.

As I mentioned in the Introduction, I intend this article to be more descriptive than prescriptive. Although I hope that some of the practices I describe above will prove useful for many readers, the most important take-home message is that the logistics of efficiently performing accurate, reproducible computational experiments is a subject worthy of consideration and discussion. Many relevant topics have not been covered here, including good coding practices, methods for automation of experiments, the logistics of writing a manuscript based on your experimental results, etc. I therefore encourage interested readers to post comments, suggestions, and critiques via the *PLoS Computational Biology* Web site.
